# Targeting acetyl-CoA carboxylases for the treatment of MASLD

**DOI:** 10.1016/j.jlr.2024.100676

**Published:** 2024-10-25

**Authors:** María Antonia Mateo-Marín, Michele Alves-Bezerra

**Affiliations:** Department of Biomedicine, Biotechnology and Public Health, Biomedical Research and Innovation Institute of Cadiz (INiBICA), Faculty of Medicine, University of Cadiz, Cadiz, Spain

**Keywords:** de novo lipogenesis, DNL, lipid metabolism, MASLD, hepatic steatosis, DNL inhibitors, ACC inhibitors

## Abstract

Hepatic accumulation of triglycerides is a hallmark feature of metabolic dysfunction-associated steatotic liver disease (MASLD). Growing evidence indicates that increased rates of de novo lipogenesis (DNL) are one of the earliest metabolic changes promoting hepatic steatosis in the onset of MASLD. The first step in DNL is catalyzed by acetyl-CoA carboxylases (ACC), which mediate the conversion of acetyl-CoA into malonyl-CoA. Given the critical role of ACC enzymes on DNL, ACC-based therapies have emerged as an attractive approach to address MASLD, leading to the development of pharmacologic inhibitors of ACC. In clinical trials, several of those compounds led to decreased DNL rates and improved hepatic steatosis in patients with MASLD. In this review, we describe the development of ACC dual inhibitors and isoform-specific inhibitors along with their clinical testing using monotherapy and combination therapy approaches. We also discuss their efficacy and safety profiles, identifying potential directions for future research. It is anticipated that advances in ACC-based therapies will be critical to the management of MASLD.

Over the last decades, significant changes in lifestyle have paralleled the rise of obesity and metabolic diseases. In this context, metabolic dysfunction-associated steatotic liver disease (MASLD; formerly known as non-alcoholic fatty liver disease, NAFLD) (1) has emerged as a global epidemic, with a worldwide prevalence of ∼32% among the adult population (2).

MASLD encompasses a spectrum of histopathologies from simple hepatic steatosis to metabolic dysfunction-associated steatohepatitis (MASH; formerly non-alcoholic steatohepatitis, NASH) (1), in which steatosis is accompanied by inflammation, hepatocyte ballooning, and often fibrosis (3). MASLD may also progress to severe pathologies such as cirrhosis (4) and hepatocellular carcinoma (HCC) (5). In addition, MASLD has long been considered the hepatic manifestation of metabolic syndrome (6, 7, 8, 9). As such, patients with MASLD are at increased risk of obesity, type 2 diabetes, and dyslipidemia. MASLD prevalence and severity can also be influenced by genetic variants, such as PNPLA3, TM6SF2, and HSD17B13 (10). Yet, the complete understanding of MASLD’s natural progression remains incomplete, therefore limiting clinical and therapeutic advances for MASLD treatment (11).

The hallmark feature of MASLD is hepatic steatosis, which is characterized by an excessive accumulation (greater than 5% of liver weight) of intrahepatic triglyceride (IHTG) (12, 13). Human isotope-labeling studies have demonstrated that the majority of hepatic triglyceride (TG) in MASLD comes from circulating fatty acids (FA), whose levels are often increased in the setting of obesity and insulin resistance (14, 15). In recent years, increased rates of hepatic de novo lipogenesis (DNL) have also emerged as a key metabolic characteristic of patients with MASLD and a significant contributor to hepatic TG accumulation in the context of the disease (14, 16, 17, 18). Hepatic DNL is the biochemical pathway for FA synthesis from acetyl-coenzyme A (CoA) substrate (19). The first step in DNL is catalyzed by acetyl-CoA carboxylases (ACC), which mediate the conversion of acetyl-CoA into malonyl-CoA (20). In mammals, two isoforms of ACC enzyme exist—ACC1, which is a cytosolic enzyme predominantly expressed in lipogenic tissues, such as adipose tissue, mammary gland, and liver, and ACC2, which localizes to the outer mitochondrial membrane and is primarily expressed in highly oxidative tissues, including skeletal muscle and heart (21, 22).

Seminal studies using ACC knockout (KO) mouse models and antisense oligonucleotides (ASO)-treated mice revealed distinct metabolic roles for ACC isoforms. Whereas malonyl-CoA produced by ACC1 is a substrate committed to the DNL pathway, thereby contributing to the synthesis of new FA molecules, malonyl-CoA produced by ACC2 primarily acts on the negative regulation of fatty acid β-oxidation (FAO) (23, 24). This is because ACC2-produced malonyl CoA inhibits the carnitine palmitoyltransferase 1 (CPT1) (25), thereby limiting the entry of FA substrates into the mitochondria for oxidation (26, 27) (Fig. 1).

**Fig. 1:**
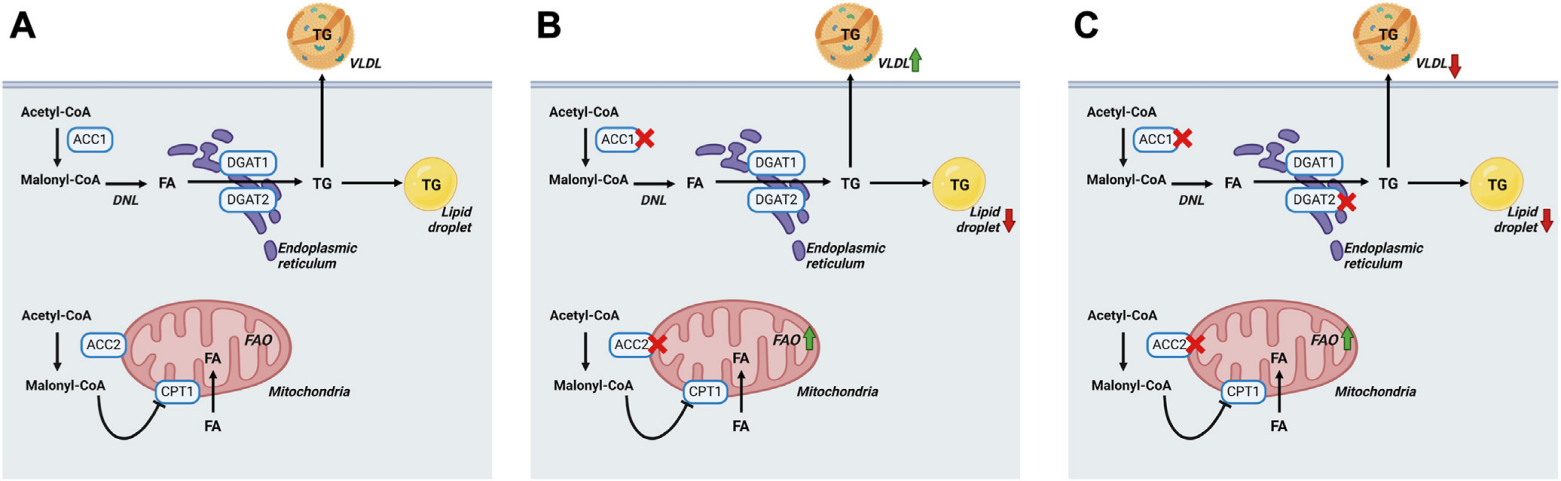
ACC1 and ACC2 inhibition strategies are currently under investigation for the treatment of MASLD. A: Acetyl carboxylase 1 (ACC1) is a cytosolic enzyme committed to de novo lipogenesis (DNL). De novo synthesized fatty acids (FA) can be channeled to triglyceride (TG) synthesis for storage within lipid droplets or for secretion into the plasma within very-low-density lipoprotein (VLDL) particles. ACC2 is bound to the outer mitochondrial membrane and produces malonyl-CoA that inhibits the carnitine palmitoyltransferase 1 (CPT1), thereby limiting the entry of FA substrates into the mitochondria for FA oxidation (FAO). B: the dual inhibition of ACC1 and ACC2 (ACC1/2) improves hepatic steatosis in patients with MASLD, likely due to decreased incorporation of TG into lipid droplets and increased FAO. However, this is often accompanied by hypertriglyceridemia, a side-effect likely linked to the dysregulation of polyunsaturated fatty acids (PUFA) and the SREBP1c pathway. C: combination therapy using ACC1/2 inhibitors co-administered with anti-lipidemic drugs, such as diacylglycerol acyltransferase 2 (DGAT2) inhibitors, are effective in improving MASLD and mitigating the adverse effect of hypertriglyceridemia caused by ACC1/2 inhibitors.

Given the critical roles of ACC enzymes on lipid homeostasis, ACC-based therapies have emerged as an attractive approach to address MASLD (28). Experimental treatment of patients with MASLD using ACC inhibitors (ACCi) resulted in decreased rates of hepatic DNL and, consequently, marked reductions in hepatic steatosis (29).

Considering the clinical relevance of ACC-based therapies, this review aims to discuss recent advances in preclinical and clinical trials employing ACCi for the treatment of MASLD. We also review potential risks associated with the use of ACCi and current efforts to improve treatment outcomes. Additional therapies targeting distinct DNL enzymes, such as fatty acid synthase (FAS) and stearoyl-CoA desaturase (SCD), have been reviewed elsewhere (30).

## Changes in DNL Regulation in MASLD

In healthy lean individuals in the fasting state, the relative contribution of DNL toward the liver lipid pool is approximately 10%. However, in obese individuals with healthy livers, these rates increase to 10%–20%, while in patients with MASLD it reaches 25%–40% (14, 16, 17, 31).

The control of DNL is primarily transcriptional. Plasma insulin activates the endoplasmic reticulum membrane-bound transcription factor sterol regulatory element binding protein 1C (SREBP1c), the N-terminus of which translocates to the nucleus and upregulates all genes in the FA biosynthetic pathway (32). In addition, the hepatic uptake of excess plasma glucose promotes the nuclear translocation of carbohydrate response element binding protein (ChREBP), a transcription factor that also upregulates transcription of the majority of FA biosynthetic genes plus pyruvate kinase (33, 34), which increases the availability of citrate for FA synthesis. In agreement with that, SREBP1c and key DNL enzymes were found to be upregulated at the transcriptional level in patients with MASLD as well as in cell culture and animal models of MASLD (35, 36). However, data on the transcriptional regulation of ACC isoforms in patients with MASLD is scarce. In a study performed in a small cohort of patients with MASLD, Kohjima *et al.* (37) reported an increase in the hepatic expression levels of *ACACA* (which encodes ACC1), along with unchanged levels of *ACACB* (ACC2-encoding gene) (37). Similarly, Mitsuyoshi *et al.* (38) found increased levels of *ACACA* gene expression in patients with MASLD with simple steatosis and MASH, whereas *ACACB* mRNA levels were not accessed in this study (38).

In addition to changes at the transcriptional level, high glucose diets were found to stimulate FA synthesis in healthy adult subjects as determined by isotope-labeling techniques (39). Moreover, recent studies indicate that the daily intake of sugar-sweetened beverages (SSBs) may also contribute to the dysregulation of DNL and the pathogenesis of MASLD in both adult and youth populations (40, 41). The consumption of SSBs has increased dramatically in the last decades, with most commercially available beverages sweetened with high-fructose corn syrup in the United States or sucrose (a disaccharide containing fructose and glucose) in European countries. Evidence exists that fructose intake may promote metabolic disturbances in the liver more effectively than glucose via upregulation of lipogenic enzymes in the DNL pathway, including *ACACA* and fatty acid synthase (*FASN*) gene (42, 43). In line with that, it was shown that the daily consumption of moderate amounts (80 g/day, comparable to amounts provided by commercial soft drinks and fruit juices) of either fructose or sucrose, but not glucose, increases hepatic DNL in healthy individuals (40). Thus, fructose found in SSBs is now considered a potent stimulator of hepatic DNL and its intake could contribute to the increasing prevalence of MASLD in Western countries in recent years. This most likely involves the hepatic upregulation of ACC-encoding genes.

Although multiple factors associated with overnutrition and obesity are known to trigger changes in DNL, further investigation is required to elucidate the molecular mechanisms leading to increased DNL specifically in patients with MASLD, and whether this involves the differential regulation of each ACC isoform.

## Dual Inhibitors of ACC1/2 in the Management of MASLD

The standard treatment for MASLD is centered on improving lifestyle to achieve better physical fitness and weight loss. In March 2024, the FDA approved the use of Resmetirom, a thyroid hormone receptor-β (THR-β) agonist, as the first and only medication for the treatment of adults with noncirrhotic MASH with moderate to advanced liver fibrosis (fibrosis stages F2 to F3) (44), in conjunction with healthy diet and exercise. It has yet to be confirmed whether Resmetirom could be used for the treatment of other MASLD stages.

In the last decade, the growing understanding of DNL's contribution to the pathogenesis of MASLD has led to the development and clinical testing of numerous DNL inhibitors as potential therapies for the management of this disease. In particular, the pharmacological inhibition of ACC enzymes represents a promising therapeutic strategy for addressing MASLD due to its potential to simultaneously inhibit FA synthesis and stimulate FAO (45). While some compounds have failed, others exhibit promising therapeutic effects in ongoing clinical trials (Table 1).

**Table 1:** Clinical trials investigating the use of ACC inhibitors for the treatment of MASLD

Drug	Function	Monotherapy/Combination therapy	Clinical improvement^a^			Study phase	Clinical trial identifier^b^
			Steatosis	MASH	Fibrosis		
PF-05175157	Dual ACC1/2 inhibitor	Monotherapy	-	-	-	I	NCT01433380
PF-05175157	Dual ACC1/2 inhibitor	Monotherapy	-	-	-	I	NCT01537497
Clesacostat (PF-05221304)	Dual ACC1/2 inhibitor	Monotherapy	-	-	-	I	NCT02871037
Clesacostat (PF-05221304)	Dual ACC1/2 inhibitor	Monotherapy	-	-	-	I	NCT03597217
Clesacostat (PF-05221304)	Dual ACC1/2 inhibitor	Monotherapy	-	-	-	I	NCT04395950
Clesacostat (PF-05221304)	Dual ACC1/2 inhibitor	Monotherapy	Yes	-	-	II	NCT03248882
Clesacostat (PF-05221304)	Dual ACC1/2 inhibitor	Co-administration with PF-06865571 (DGAT2 inhibitor)	-	-	-	II	NCT04321031
Clesacostat (PF-05221304)	Dual ACC1/2 inhibitor	Co-administration with PF-06865571 (DGAT2 inhibitor)	Yes	-	-	II	NCT03776175
Firsocostat (GS-0976)	Dual ACC1/2 inhibitor	Monotherapy	-	-	-	I	NCT02891408
Firsocostat (GS-0976)	Dual ACC1/2 inhibitor	Monotherapy	Yes	-	Yes	II	NCT02856555
Firsocostat (GS-0976)	Dual ACC1/2 inhibitor	Co-administration with Semaglutide, Firsocostat, and/or Cilofexor	-	-	-	II	NCT04971785
Firsocostat (GS-0976)	Dual ACC1/2 inhibitor	Co-administration with Semaglutide, Firsocostat, and/or Cilofexor	Yes	Yes	-	II	NCT03987074
Firsocostat (GS-0976)	Dual ACC1/2 inhibitor	Co-administration with Selonsertib, Firsocostat, and/or Cilofexor	Yes	Yes	Yes	II	NCT03449446
Firsocostat (GS-0976)	Dual ACC1/2 inhibitor	Co-administration with Selonsertib, Firsocostat, Cilofexor, Fenofibrate, and/or Vascepa	Yes	-	-	II	NCT02781584
MK-4074	Dual ACC1/2 inhibitor	Monotherapy	Yes	-	-	I	NCT01431521

a The symbol "-" denotes the lack of specific information.

b Registration number in ClinicalTrials.gov.

The first generation of ACC inhibitors included Pfizer's PF-05175157, a potent and reversible dual inhibitor of ACC1 and ACC2 (ACC1/2) with systemic distribution. In a phase I clinical trial, it was observed to inhibit hepatic DNL in a dose- and time-dependent manner (46). Single doses of PF-05175157 were generally well tolerated. However, participants administered the highest repeated dose (200 mg twice daily for 14 days) exhibited time-dependent, asymptomatic decreases in platelet count, likely due to DNL impairment in the bone marrow. Although platelet counting reductions were quickly reversed after treatment interruption (47), this safety concern led to the discontinuation of clinical trials involving PF-05175157.

Currently available ACC1/2 dual inhibitors, such as MSD's MK-4074 (48), Gilead's GS0976, also known as Firsocostat (45, 49), and Pfizer's PF-05221304, also known as Clesacostat (50, 51), showed great efficacy in inhibiting hepatic DNL. Kim *et al.* (48) were among the first groups to transition from preclinical animal models to clinical evaluation of ACCi. Their findings revealed that the administration of MK-4074 (200 mg twice daily for 4 weeks) led to a marked reduction (36% decrease) in intrahepatic fat in patients with hepatic steatosis. Nonetheless, it also resulted in an elevation in mean plasma TG concentrations, encompassing TG associated with very low-density lipoproteins (VLDL), low-density lipoproteins (LDL), and high-density lipoproteins (HDL) (48).

In a phase 2 randomized placebo-controlled trial targeting patients with MASH, Loomba *et al.* (52) investigated the efficacy of GS-0976. A 12-week therapy with GS-0976 (20 mg daily) led to significant improvements in hepatic steatosis and markers of fibrosis (52). Their findings are in line with a later study showing that a 12-week administration of GS-0976 (20 mg daily) resulted in a 22% median decrease in hepatic DNL rates in MASH patients compared to baseline, along with decreased steatosis and a reduction in markers of liver injury (29). However, 8%–20% of individuals treated with GS-0976 also experienced a notable surge in serum TG levels, prompting the withdrawal of participants from these clinical trials (45, 52). Similar results were obtained for PF-05221304. Whereas the administration of PF-05221304 (2, 10, 25 or 50 mg daily) resulted in a significant anti-steatotic impact and improvements in certain MASH-related biomarkers over a 16-week follow-up period, the observed elevations in serum TG raised concern about increased cardiometabolic risk upon intervention, thereby restricting the long-term utility of this treatment (51).

The mechanistic basis of ACCi-associated hypertriglyceridemia was further investigated in mice (48). In this model, the simultaneous liver-specific deletion of ACC1 and ACC2-encoding genes resulted in a relative deficiency of polyunsaturated fatty acids (PUFAs) in the liver, which led to the activation of SREBP1c and increased expression of the genes encoding glycerol-3-phosphate acyltransferase 1 (GPAT1), an enzyme involved in TG synthesis, and patatin-like phospholipase domain-containing protein 3 (PNPLA3), a TG lipase that may promote the hepatic secretion of VLDL (48, 53, 54). Both PUFA supplementation and siRNA-mediated knockdown of GPAT1 were able to restore normal plasma TG levels in mice lacking ACC1/2 (48). However, it remains uncertain whether the PUFA depletion and SREBP1c activation reported in mice also contribute to hypertriglyceridemia in patients treated with ACC1/2 dual inhibitors.

Combination therapy approaches have been tested to circumvent the concerns associated with ACCi-induced hypertriglyceridemia. A phase 2a trial was used to investigate the effects of liver-directed dual inhibition of ACC1/2 using PF-05221304 co-administered with PF-06865571, a diacylglycerol acyltransferase 2 (DGAT2) inhibitor (51). DGAT enzymes catalyze the final step in TG synthesis through the esterification of a fatty acyl-CoA into diacylglycerol (DG), thus producing TG (55). Whereas the sole administration of the ACCi PF-05221304 in individuals with MASLD resulted in decreased hepatic fat accumulation and liver injury, it also led to a significant rise in serum TG levels as previously reported. However, the co-administration of the DGAT2 inhibitor PF-06865571 effectively mitigated hypertriglyceridemia during a 6-week follow-up period (51).

Combination therapy using GS-0976 and Fenofibrate also exhibited remarkable results in clinical trials for MASLD/MASH (56, 57). Fenofibrate, a peroxisome proliferator-activated receptor α (PPAR-α) agonist, is extensively used in clinical practice for managing dyslipidemia (58) as it reduces hepatic TG levels, although the mechanism underlying this effect in human subjects remains incompletely understood. While improving MASLD/MASH with GS-0976, co-administration with Fenofibrate for 6–24 weeks alleviated the side-effect of hypertriglyceridemia in those patients (56, 57).

Although ACC1/ACC2 dual inhibitors have been effective for the management of MASLD in the preclinical setting, the associated hypertriglyceridemia often observed in ACCi-based therapies represents a critical limitation for its clinical implementation. The causes of dyslipidemia in ACCi-treated patients are not fully understood and require further mechanistic studies. In this context, combination therapy emerged as an alternative strategy to reduce the adverse effects of ACCi administration in the short term; however, it remains to be confirmed whether combination therapies would show efficacy in prolonged treatment durations.

## Selective Inhibitors of ACC1 and ACC2

Currently, no registered clinical trials are testing selective inhibitors for individual ACC isoforms. However, several studies have provided insights from animal models. In mice, it has been reported that ACC1 global KO in the germline leads to embryonic lethality (59). To circumvent this limitation, ACC1 liver-specific knockout (L-KO) mice were created. ACC1 L-KO mice generated by Mao *et al.* (23) showed a significant reduction in DNL and TG synthesis in the liver when fed a Chow diet (approximately 40% less than in wild-type, WT, mice). Despite this reduction, plasma TG levels in lean ACC1 L-KO mice were similar to those observed in WT mice. Fasting ketone body levels, a surrogate marker of FAO, also remained unchanged in chow-fed ACC1 L-KO mice (23). Nonetheless, when fed a high-fat, high-carbohydrate (HF/HC) diet, ACC1 L-KO mice were not protected against obesity, fatty liver, and diabetes. Under this specific lipogenic diet, both WT and ACC1 L-KO mice showed similar body weights and similar levels of TG accumulation. Additionally, both genotypes exhibited glucose intolerance and insulin resistance under HF/HC feeding. (23).

By contrast, ACC1 L-KO mice generated by Harada *et al.* (60) were protected against hepatic steatosis induced by a high-sucrose diet (HSD) feeding. Liver TG contents in the HSD-fed ACC1 L-KO mice were decreased by approximately 70% compared to that in the HSD-fed control genotype mice. This is likely attributable to the observed 25% decrease in hepatic DNL rates in the HSD-fed ACC1 L-KO mice compared to their counterparts (60).

ACC2 null mice were generated by independent laboratories with conflicting phenotypic data. Whole-body ACC2 KO mice generated by Olson *et al.* (61) displayed minimal changes in fat metabolism compared to control animals when fed either a regular chow or a high-fat diet (HFD) (61). However, ACC2 null mice described by Abu-Elheiga *et al.* (62) exhibited increased rates of FAO in muscle and heart, leading to reduced body adiposity (22). When fed an HF/HC diet, ACC2 KO mice also exhibited higher serum levels of ketone bodies compared to HF/HC-fed WT mice (62). Interestingly, serum insulin levels were lower in ACC2 whole-body KO mice fed an HF/HC diet compared to their WT counterparts (62). Furthermore, Choi *et al.* (63) found that fasting plasma glucose and insulin concentrations were decreased by 30% in HFD whole-body ACC2 KO mice compared to HFD WT mice, while plasma FA, TG, and cholesterol levels remained similar in both groups (63).

Although informative, studies with KO animal models are not ideal for predicting the therapeutic outcomes of a specific target blockade. This is because, in these models, the loss of the target protein occurs from birth and persists throughout the lifespan. Additionally, KO animal models are based on the complete loss of a target, which is in contrast with therapeutic approaches that may lead to partial inhibitions. Hence, the use of pharmacological or genetic inhibitors is arguably a more reliable preclinical approximation. In a preclinical study, Compound-1, an ACC1-selective inhibitor, was tested for the treatment of diet-induced MASLD in a murine model (64). Whereas Compound-1 administration resulted in remarkable decreases in IHTG accumulation, it retained the adverse effect of hypertriglyceridemia (64) as observed for ACC1/2 dual inhibitors (59).

There is scarce data available on ACC2-specific inhibitors. The administration of ACC2-selective inhibitors (S enantiomer of compound 9c ([S]-9c) and Compound 2e) to mice and rats was shown to reduce malonyl-CoA concentrations and to stimulate FAO in skeletal muscle (65, 66). However, these studies were centered on the effects of ACC2-inhibition on muscle physiology. To our knowledge, there are no studies reporting the effect of pharmacological inhibition of ACC2 on liver metabolism and/or MASLD.

Because ACC1/2 dual inhibitors and ACC1-specific inhibitors are associated with MASLD improvement along with the adverse effect of hypertriglyceridemia, it is tempting to speculate that ACC2-specific inhibitors could lead to increased FAO rates and, thereby, improved MASLD in the absence of dyslipidemia. However, additional studies are required to clarify the effects of the ACC2 blockade and whether this could be explored in the context of MASLD.

## Conclusion and Outlook

ACC1/2 dual inhibitors have emerged as leading lipogenesis modulators for the treatment of MASLD due to their robust effects in DNL rates and IHTG levels in patients with MASLD (Table 1). Although promising, ACCi-based therapies also present important limitations: To date, studies on DNL rates in human subjects are limited to small cohorts and it remains unclear whether DNL dysregulation is a broad feature for patients with MASLD or a specific characteristic of subsets of patients. In addition, it is currently unknown the relative contribution of distinct ACC isoforms towards the increased DNL rates reported in obese subjects and patients with MASLD. These limitations could be addressed by improving clinical studies to include large and more diverse populations.

Further, the use of ACC1/2 dual inhibitors has been associated with concerning side effects, including hypertriglyceridemia and reduced platelet count. In recent years, combination therapy (including the coadministration of ACCi and DGAT inhibitors or ACCi and Fenofibrate) has improved the safety profile for ACCi-based therapies. However, these studies are still limited to small cohorts and short-term interventions. It is anticipated that larger clinical trials will improve our understanding of the combinatorial administration of ACCi and other therapeutic agents.

Finally, previous research was centered on the use of ACC1/2 dual inhibitors, with limited information on the therapeutic potential of isoform-specific inhibitors. Preclinical data suggests that ACC1-specific inhibitors are effective in decreasing DNL rates and limiting IHTG. However, it appears to retain the adverse effect of hypertriglyceridemia observed upon ACC1/2 dual inhibitor administration. Nonetheless, the effect of ACC1-specific inhibitors is yet to be confirmed in human subjects. Based on studies performed in animal models, it is tempting to speculate that ACC2-specific inhibitors could increase FAO rates and, therefore, limit IHTG in the absence of hypertriglyceridemia. Further preclinical and clinical studies will help to address this possibility and guide the development of novel treatment options for MASLD.

## Data availability

All data supporting this study are included in the manuscript.

## Conflict of interest

The authors declare that they have no conflicts of interest with the contents of this article.
